# Time trends in depression prevalence among Swedish 85-year-olds: repeated cross-sectional population-based studies in 1986, 2008, and 2015

**DOI:** 10.1017/S0033291721004335

**Published:** 2023-04

**Authors:** Mattias Jonson, Robert Sigström, Khedidja Hedna, Therese Rydberg Sterner, Hanna Falk Erhag, Hanna Wetterberg, Madeleine Mellqvist Fässberg, Margda Waern, Ingmar Skoog

**Affiliations:** 1 Department of Psychiatry and Neurochemistry, Center for Ageing and Health (Age Cap), Gothenburg University, Gothenburg, Sweden; 2 Neuropsychiatric Epidemiology Unit, Department of Psychiatry and Neurochemistry, Sahlgrenska Academy, University of Gothenburg, Gothenburg, Sweden; 3 Region Västra Götaland, Sahlgrenska University Hospital, Affective Clinic, Gothenburg, Sweden; 4 Region Västra Götaland, Sahlgrenska University Hospital, Clinic of Cognition and Old Age Psychiatry, Gothenburg, Sweden; 5 Statistikkonsulterna Jostat & Mr Sample AB, Gothenburg, Sweden; 6 Region Västra Götaland, Sahlgrenska University Hospital, Psychosis Clinic, Gothenburg, Sweden

**Keywords:** Psychiatric epidemiology, old age psychiatry, cohort study, late life depression, pharmacoepidemiology, gerontology

## Abstract

**Background:**

Octogenarians of today are better educated, and physically and cognitively healthier, than earlier born cohorts. Less is known about time trends in mental health in this age group. We aimed to study time trends in the prevalence of depression and psychotropic drug use among Swedish 85-year-olds.

**Methods:**

We derived data from interviews with 85-year-olds in 1986–1987 (*N* = 348), 2008–2010 (*N* = 433) and 2015–17 (*N* = 321). Depression diagnoses were made according to the Diagnostic and Statistical Manual of Mental Disorders. Symptom burden was assessed with the Montgomery-Åsberg Depression Rating Scale (MADRS). Information on psychotropic drug use, sociodemographic, and health-related factors were collected during the interviews.

**Results:**

The prevalence of major depression was lower in 2015–2017 (4.7%, *p* < 0.001) and 2008–2010 (6.9%, *p* = 0.010) compared to 1986–1987 (12.4%). The prevalence of minor depression was lower in 2015–2017 (8.1%) compared to 2008–2010 (16.2%, *p* = 0.001) and 1986–1987 (17.8%, *p* < 0.001). Mean MADRS score decreased from 8.0 in 1986–1987 to 6.5 in 2008–2010, and 5.1 in 2015–2017 (*p* < 0.001). The reduced prevalence of depression was not explained by changes in sociodemographic and health-related risk factors for depression. While psychoactive drug use was observed in a third of the participants in each cohort, drug type changed over time (increased use of antidepressants and decreased use of anxiolytics and antipsychotics).

**Conclusions:**

The prevalence of depression in octogenarians has declined during the past decades. The decline was not explained by changes in known risk factors for depression. The present study cannot answer whether changed prescription patterns of psychoactive drugs have contributed to the decline.

## Introduction

The continuously increasing life expectancy worldwide reflects how changes in environmental exposures affect people's health across the lifespan (Christensen, Doblhammer, Rau, & Vaupel, 2009; Vaupel, 2010). Previous reports show improved physical health (Horder, Skoog, Johansson, Falk, & Frandin, 2015; Wilhelmson, Allebeck, & Steen, 2002) and increased medication usage (Craftman, Johnell, Fastbom, Westerbotn, & von Strauss, 2016) in older adults. Further, evidence suggests that the incidence and prevalence of dementia are on the decline in Western countries (Wu et al., 2017), which may be related to increased educational level (Skoog et al., 2017) and thereby an increased cognitive reserve (Stern, 2012). However, research about trends in the prevalence of mental disorders in older adults without dementia is relatively scarce. As depression in older adults is associated with physical health (Djernes, 2006), an improvement in the latter might predict an improvement in the former.

There are several approaches to study time trends in mental health; one is to utilize administrative data. Studies from the United States based on such data have reported increases in depression diagnoses in older adults during the past decades (Akincigil et al., 2011; Akushevich, Kravchenko, Yashkin, & Yashin, 2018). In contrast, the same type of studies from Great Britain found reductions in depression diagnoses in older adults during a similar period (Kendrick, Stuart, Newell, Geraghty, & Moore, 2015; Rait et al., 2009). In parallel, antidepressant prescriptions nearly doubled in Britain between 1993 and 2005 (Moore et al., 2009). It is important to note that trends based on this type of data may reflect changes in detection rates as well as the availability of treatments suitable for older adults (Dolder, Nelson, & McKinsey, 2007; Wilson & Mottram, 2004), rather than actual changes in the mental health of the populations under study (Christensen et al., 2009).

Another approach in the study of time trends is to estimate trends by a systematic review of studies conducted at different time points. The Global Burden of Disease Report estimated that global age-standardized prevalences of depressive disorders between 1990 and 2017 were stable (Liu et al., 2020). A narrative review of studies from several high-income countries found no decline in the prevalence of common mental disorders in the last decades, despite increases in spending on mental health care and antidepressant use (Jorm, Patten, Brugha, & Mojtabai, 2017).

As methodological differences may hamper the comparison of studies conducted at different time points, repeated studies of comparable population-based samples, using identical methods, may yield better estimates of potential time trends (Christensen et al., 2009). Such studies from Australia (Hawthorne, Goldney, & Taylor, 2008), Great Britain (Arthur et al., 2020), and Sweden (Liang, Rausch, Laflamme, & Möller, 2018) found no differences in the prevalence of depression at different time points. Contrasting this, a recent study from our group reported a reduced prevalence of depression among 70-year-old women between 1976 and 2016 (Rydberg Sterner et al., 2019). Population-based data from 18 European countries showed decreased depression scores in older adults (Beller et al., 2021). Similarly, declining depression symptom burden was reported in two US-based studies (Sullivan et al., 2020; Zivin, Pirraglia, McCammon, Langa, & Vijan, 2013), and in one of these (Zivin et al., 2013), symptom reduction was driven by changes in those aged 80–84.

Since old age encompasses a broad age segment, in which time trends might differ, it is of importance to investigate time trends in specific segments of older adults. We aimed to study time trends in the prevalence of major and minor depression, depression symptom score, and psychotropic drug use in 85-year-olds living in Gothenburg, Sweden. We hypothesized that we would observe a decrease in depression prevalence and that changes in known sociodemographic and health-related risk factors for depression would help to explain that decrease.

## Methods

### Samples

Population samples of 85-year-olds born in 1901–1902, 1923–1924, and 1930 were examined in 1986–1987, 2008–2010, and 2015–2017, respectively. For simplicity, the cohorts will henceforth be referred to as 1986, 2008, and 2015. All samples were systematically obtained from the Swedish Population Register, based on birth date. The samples included persons living in both private households and long-term care facilities. For this paper, participants with Mini-Mental State Examination (MMSE) (Folstein, Folstein, & McHugh, 1975) score of 23 points or lower (Trivedi, 2017) were excluded, as were those lacking MMSE ratings.

In 1986, all psychiatric examinations were carried out in the participants' homes. In 2008 and 2015, participants were invited to an outpatient clinic. Those who declined or were not able to visit the clinic were offered home visits. Examinations were performed by a psychiatrist in 1986, by psychiatric research nurses in 2008, and by psychiatric research nurses or medical doctors in 2015. Those performing the examinations in 2008 and 2015 were trained and supervised by the co-last author (I.S.), who performed all the examinations in 1986.

### Cohort 1986

In 1986, 494 individuals (143 men and 351 women) participated (response rate 64.2%). Non-participants and participants did not differ regarding sex or 3-year survival rate (71.3% *v.* 74.1%) (Skoog, Nilsson, Palmertz, Andreasson, & Svanborg, 1993). We excluded 143 participants due to low MMSE scores and three due to missing MMSE data, leaving 348 participants (108 men and 240 women).

### Cohort 2008

In 2008, 571 (212 men and 359 women) participated (response rate 60.5%). Participants and non-participants did not differ regarding sex. Non-participants had a lower 3-year survival rate (76.7% *v.* 83.4%, *p* = 0.011) (Skoog et al., 2017). We excluded 122 participants due to low MMSE scores and 16 due to missing MMSE data, leaving 433 participants (163 men and 270 women).

### Cohort 2015

In 2015, 416 (165 men and 251 women) participated (response rate 61.9%). Participants and non-participants did not differ regarding sex. Non-participants had a lower 3-year survival rate (76.6% *v.* 83.1%, *p* = 0.027). We excluded 89 participants due to low MMSE scores, five due to missing MMSE data, and one who did not complete the psychiatric interview, leaving 321 participants (129 men and 192 women).

### Assessments

#### Diagnostic procedures

Psychiatric symptoms and signs were assessed using the semi-structured Comprehensive Psychopathological Rating Scale (CPRS) (Åsberg, Montgomery, Perris, Schalling, & Sedvall, 1978). Each item is rated on a six-point scale. A computerized algorithm based on CPRS items was employed to establish whether participants met diagnostic criteria for major depression. The algorithm was designed to follow the definition of major depressive episode in the Diagnostic and Statistical Manual for Mental Disorders, Fifth Edition (DSM-5) (American Psychiatric Association, 2013) as closely as possible: At least five of the nine depressive symptoms or signs listed in DSM-5 had to be present, at least one of which had to be either depressed mood or loss of interest (Sigström, Waern, Gudmundsson, Skoog, & Östling, 2018). Exclusion criteria for depression could not be applied due to the difficulty of making etiological judgments in an epidemiological study. Since non-major depression may be of clinical relevance in older adults (Meeks, Vahia, Lavretsky, Kulkarni, & Jeste, 2011), we also applied the research criteria of minor depression proposed in the revision of DSM-IV (American Psychiatric Association, 2000): At least two, but less than five depressive symptoms or signs, at least one of which had to be either depressed mood or loss of interest. Additionally, a dimensional approach was employed; symptoms of depression were rated with the Montgomery-Åsberg Depression Rating Scale (MADRS) (Montgomery & Asberg, 1979), a 10-item scale derived from the CPRS.

Symptoms and signs employed in the depression algorithm were assessed with dual ratings by research nurses and psychiatrists in 169 individuals, from all of the Gothenburg H70 Birth Cohort Studies. On average there was a substantial agreement (*κ* = 0.63), ranging from fair (*κ* = 0.39) for psychomotor retardation to very good (*κ* = 0.85) for hypersomnia (Supplementary Table S1).

#### Psychotropic drug use

Information on the current use of psychotropic drugs was collected, in a standardized manner, during the interviews: The interviewer asked the participant what medications they were currently taking and/or reviewed medication lists provided by the participant. Each medication was classified according to the Anatomic Therapeutic Chemical Classification System (ATC) as an antidepressant (N06A), anxiolytic (N05B), sedative or hypnotic (N05C), or antipsychotic (N05A) (World Health Organization, 2013). Lithium (classified as an antipsychotic in the ATC) and alimemazine (classified as an antipsychotic in 1986, but later re-classified as an anti-allergic drug) were not included in any of these categories but were included in psychotropic drugs total. Other drugs with possible psychiatric indications (e.g. anti-epileptic drugs) were not included in the estimate because of a high likelihood of non-psychiatric indication. We lacked comparable cohort-specific information regarding dosage (daily or as needed) and indications.

#### Sociodemographic factors

Self-reported information on educational level [mandatory *v.* more than mandatory (>6 years for cohorts 1986 and 2008, >7 years for cohort 2015)], type of residence (private household *v.* long-term care), partner loss during the last 5 years, currently having a partner or not, and feelings of loneliness (never, rarely or sometimes feeling lonely *v.* often feeling lonely) were collected during the face-to-face interview.

#### Health-related factors

A single question was asked regarding the participants’ perception of their own current health. Responses were dichotomized as follows: good self-rated health (good/very good) or poor self-rated health (fairly poor/very poor). Health conditions were self-reported and ascertained by a positive answer to the question: “Have you ever been told by a doctor that you have…?” In the present study, we used the following health conditions: diabetes mellitus, pulmonary disorder (chronic bronchitis, asthma or chronic obstructive pulmonary disease), angina pectoris, myocardial infarction, peptic ulcer, rheumatoid arthritis, any type of cancer, hemorrhagic or ischemic stroke, or fracture of the hip, the upper arm or the wrist. Past-month pain was rated with an item from the CPRS based on intensity and duration; responses were dichotomized as no/minimal pain (rating 0–3) *v.* pain (rating 4–6). Information on dependence in activities of daily living (ADL) was collected during the interviews, based on the Katz disability index (Katz, Downs, Cash, & Grotz, 1970), and dependence was defined as the need for assistance or surveillance by another person in bathing/showering, dressing, using the toilet, transferring indoors or eating (Falk et al., 2014).

### Statistical analysis

Cohort comparisons of categorical and continuous variables were tested for statistical significance with the Pearson Chi-square test and independent samples *t* test. To examine the effect of the cohort on the prevalence of depression and adjust for possible confounders, we used a multinomial logistic regression model to calculate odds ratios for major and minor depression in cohort 2008 and 2015 compared to 1986 as the reference category. First, we conducted bivariate analyses of all variables (model 1). Second, we estimated the effect of the cohort after the inclusion of sociodemographic factors (sex, education, type of residence, partner loss during the last 5 years, no current partner, and feelings of loneliness) (model 2). Third, we also included health-related variables (number of somatic disorders, self-rated health, past month pain, and ADL dependency) (model 3). All statistical analyses were performed with IBM SPSS Statistics for Windows (Version 25).

## Results

Table 1 presents the characteristics of the samples. The majority of participants in all cohorts were women, although this proportion decreased in successive cohorts. Proportions living in long-term care were smaller in later-born cohorts while having more than compulsory education was more common in these cohorts compared to the first. Although more participants had lost their partner during the past 5 years in later-born cohorts, larger proportions in these cohorts had a current partner compared to the first cohort. There was a considerable decrease in ADL dependence between the middle and the latest born cohort.

**Table 1.** Characteristics of participants in three population-based cohorts of 85-year-olds^a^

	1986 (*N* = 348)			2008 (*N* = 433)			2015 (*N* = 321)		
	Valid cases	*N*	%^b^	Valid cases	*N*	%^b^	Valid cases	*N*	%^b^
Female sex	348	240	69.0	433	270	62.4	321	192	59.8^c^
Long-term care residence	348	30	8.6	423	15	3.5^c^	314	7	2.2^c^
Education beyond mandatory	340	100	29.4	424	254	59.9^c^	304	170	55.9^c^
Lost partner within last 5 years	348	33	9.5	415	64	15.4^c^	320	47	14.7^c^
No current partner	347	261	75.2	427	248	58.1^c^	321	185	57.6^c^
Feelings of loneliness	347	29	8.4	420	37	8.8	318	20	6.3
Poor self-rated health	347	56	16.1	430	77	17.9	320	45	14.1
Dependence in any ADL^d^	348	67	19.3	415	69	16.6	321	19	5.9^c,e^
Past-month pain	347	92	26.5	428	95	22.2	320	84	26.3
Number of somatic disorders	348		1.2	433		1.6^c^	321		1.3^e^

a For inclusion, participants must have Mini-Mental State Examination scores ⩾24.

b Mean for number of somatic disorders.

c *p* value <00.5 for the difference in proportion (Pearson's chi-square) or mean (Independent samples *t* test) compared to cohort 1986.

d Activities of daily living (bathing or showering, feeding, toileting, transferring indoors and dressing).

e *p* value <0.05 for the difference in proportion (Pearson's chi-square) or mean (Independent samples *t* test) compared to cohort 2008.

**Fig. 1.**
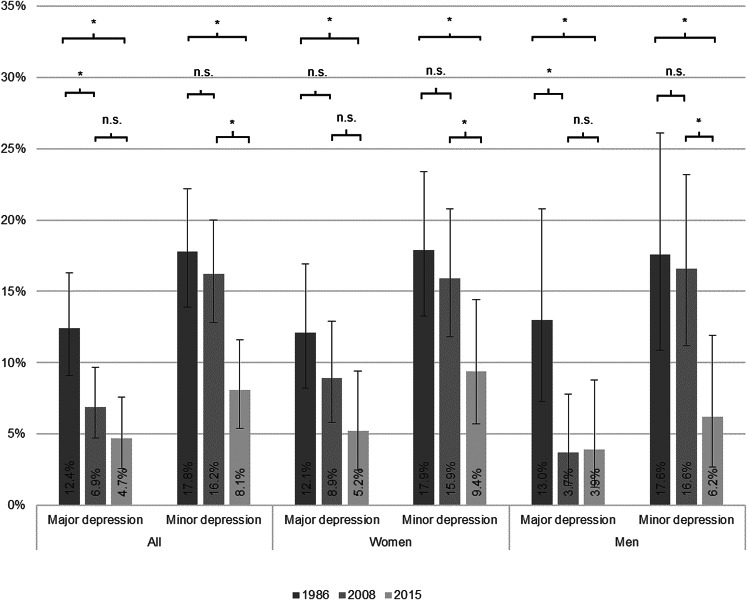
Proportions with major depression and minor depression in three population-based cohorts of 85-year-olds with MMSE score above 23, by sex. Error bars show 95% confidence intervals. * = *p* < 0.05 for the difference in proportion. n.s. = not statistically significant difference in proportion.

**Fig. 2.**
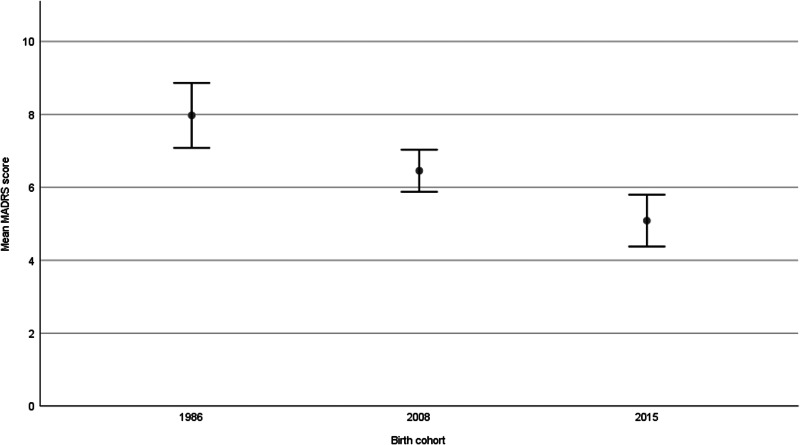
Mean MADRS-score with 95% confidence intervals in three population-based cohorts of 85-year-olds with MMSE-score above 23 (*p* = 0.004 for 1986 *v.* 2008, *p* = 0.003 for 2008 *v.* 2015, *p* < 0.001 for 1986 *v.* 2015).

As shown in Fig. 1, major depression was less common in 2008 (6.9%, *p* = 0.010) and 2015 (4.7%, *p* < 0.001) compared to 1986 (12.4%), but no difference was observed between 2008 and 2015 (*p* = 0.196). The prevalence of minor depression was similar in 1986 (17.8%) and 2008 (16.2%, *p* = 0.541) but lower in 2015 (8.1%, *p* < 0.001) compared to 1986. The decrease in major depression from 1986 to 2008 was significant for men but not for women. Comparing 1986 and 2015, the difference was significant for both sexes. The decrease in minor depression from 2008 to 2015 was significant for both sexes. As can be seen in Fig. 2, the mean MADRS score was lower in 2008 compared to 1986 (*p* = 0.004), in 2015 compared to 2008 (*p* = 0.003), and hence, in 2015 compared to 1986 (*p* < 0.001).

In bivariate analyses (Table 2), living in a long-term care facility, feelings of loneliness, poor self-rated health, ADL dependence, pain during the past month, and the number of somatic disorders were associated with major depression. The same factors were associated with minor depression, except long-term care residence and ADL dependence.

**Table 2.** Multinomial regression analysis of associations with major and minor depression in three population-based cohorts of 85-year-olds^a^ (*N* = 1102)

	Major depression (*N* = 88)			Minor depression (*N* = 158)		
	Models^b^, OR (95% Confidence interval)			Models^b^, OR (95% Confidence interval)		
	1	2	3	1	2	3
Cohort 2008^c^	0.51 (0.31–0.84)**	0.49 (0.28–0.88)*	0.37 (0.19–0.70)*	0.82 (0.56–1.20)	0.74 (0.48–1.13)	0.69 (0.44–1.08)
Cohort 2015^c^	0.30 (0.16–0.56)***	0.31 (0.16–0.63)**	0.24 (0.11–0.51)***	0.36 (0.22–0.59)***	0.32 (0.19–0.55)***	0.28 (0.16–0.49)***
Sociodemographic variables						
Female sex	1.51 (0.93–2.45)	1.80 (1.00–3.22)	1.57 (0.83–2.95)	1.16 (0.81–1.65)	1.38 (0.89–2.12)	1.20 (0.76–1.90)
Long-term care residence	2.99 (1.43–6.27)**	2.41 (1.00–5.82)	1.71 (0.60–4.86)	1.08 (0.47–2.47)	1.11 (0.46–2.67)	1.16 (0.45–3.00)
Education beyond mandatory	0.81 (0.52–1.27)	0.97 (0.58–1.61)	1.23 (0.71–2.14)	0.89 (0.63–1.26)	0.95 (0.65–1.39)	1.12 (0.75–1.66)
Lost partner within last 5 years	1.59 (0.89–2.84)	1.10 (0.53–2.29)	1.24 (0.56–2.77)	1.38 (0.86–2.21)	1.33 (0.77–2.31)	1.57 (0.89–2.79)
No current partner	1.61 (0.98–2.62)	0.57 (0.30–1.08)	0.60 (0.30–1.20)	1.04 (0.73–1.48)	0.62 (0.39–0.99)*	0.61 (0.37–0.99)*
Feelings of loneliness	14.00 (7.90–24.80)***	15.29 (7.93–29.47)***	13.22 (6.25–27.99)***	5.46 (3.13–9.5)***	5.99 (3.25–11.05)***	5.62 (2.87–11.01)***
Health-related variables						
Poor self-rated health	9.62 (5.97–15.5)***		6.91 (3.95–12.08)***	4.66 (3.11–6.97)***		3.74 (2.39–5.85)***
Dependence in any ADL	2.82 (1.68–4.72)***		1.64 (0.86–3.16)	1.54 (0.98–2.44)		1.08 (0.63–1.86)
Past-month pain	2.56 (1.62–4.06)***		1.56 (0.89–2.75)	2.29 (1.60–3.30)***		1.93 (1.27–2.93)**
Number of somatic disorders	1.23 (1.04–1.45)*		1.30 (1.05–1.60)*	1.16 (1.01–1.32)*		1.15 (0.98–1.34)

a For inclusion, participants must have Mini-Mental State Examination scores ⩾24.

b Model 1: bivariate analysis. Model 2: adjusted model including sociodemographic variables. Model 3: adjusted model including all variables.

c Reference category: Cohort 1986.

*p < 0.05, **p < 0.01, ***p < 0.001.

In a multinomial regression model (Table 2, model 1), cohort 2008 and 2015 had lower odds for having major depression than cohort 1986. Cohort 2015 had lower odds for minor depression than cohort 1986. The odds ratios did not change meaningfully when adding sociodemographic factors (model 2) and both sociodemographic and health-related factors (model 3) to the model. In model 3, major depression was associated with the cohort, feelings of loneliness, poor self-rated health, and the number of somatic disorders. A similar pattern was observed for minor depression, with the addition of no current partner and past month pain and the exception of the number of somatic disorders.

To test the robustness of our findings we reran the regression analyses after replacing the categorical dependent variable (no/minor/major depression) with the continuous MADRS-score. (Supplementary Table S2). When comparing cohort 2008 with 1986 in this manner, results were in line with those presented in Table 2. When comparing 2015 with 1986, the predictive value of cohort decreased somewhat when including sociodemographic and health-related factors, but cohort remained a significant predictor of MADRS-score.

The proportion using any psychotropic drug remained stable across cohorts (Table 3). The use of antidepressants nearly doubled between 1986 and 2008, but then remained stable. Conversely, the use of anxiolytics was about half in 2008 and 2015 compared to 1986. The use of antipsychotics dropped by more than half between 1986 and 2008. Among individuals with major depression, only the use of sedatives and hypnotics changed significantly, with higher use in 2008 and 2015 compared to 1986. Among those who fulfilled criteria for neither major nor minor depression, the use of antidepressants was more common in 2008 and 2015, compared to 1986.

**Table 3.** Use of psychotropic drugs by depression status in three cohorts of 85-year-olds^a^

	Total						Major depression						Minor depression						No depression					
	1986−198787 (*N* = 348)		2008–2010 (*N* = 433)		2015–2017 (*N* = 321)		1986–1987 (*N* = 43)		2008–2010 (*N* = 30)		2015–2017 (*N* = 15)		1986–1987 (*N* = 62)		2008–2010 (*N* = 70)		2015–2017 (*N* = 26)		1986–1987 (*N* = 243)		2008–2010 (*N* = 333)		2015–2017 (*N* = 280)	
	*n*	%	*n*	%	*n*	%	*n*	%	*n*	%	*n*	%	*n*	%	*n*	%	*n*	%	*n*	%	*n*	%	*n*	%
Antidepressants	20	5.7	52	**12.0**^b^	38	**11.8**^b^	9	20.9	10	33.3	6	40.0	2	3.2	14	**20.0**^b^	3	11.5	9	3.7	28	**8.4**^b^	29	**10.4**^b^
Anxiolytics, sedatives and hypnotics	116	33.3	136	31.4	99	30.8	24	55.8	22	73.3	10	66.7	26	41.9	28	40.0	9	34.6	66	27.2	86	25.8	80	28.6
Anxiolytics	59	17.0	34	**7.9**^b^	29	**9.0**^b^	18	41.9	11	36.7	5	33.3	16	25.8	6	**8.6**^b^	4	15.4	25	10.3	17	**5.1**^b^	20	7.1
Sedatives and hypnotics	77	22.1	115	26.6	87	27.1	11	25.6	16	**53.3**^b^	8	**53.3**^b^	16	25.8	25	35.7	5	19.2	50	20.6	74	22.2	74	26.4
Antipsychotics	19	5.5	4	**0.9**^b^	6	**1.9**^b^	9	20.9	0	**0.0**^b^	1	6.7	5	8.1	1	1.4	0	0.0	5	2.1	3	0.9	5	1.8
Any psychotropic drug	130	37.4	159	36.7	114	35.5	28	65.1	26	**86.7**^b^	10	66.7	29	46.8	35	50.0	11	42.3	73	30.0	98	29.4	93	33.2

a For inclusion, participants must have Mini-Mental State Examination scores ⩾24.

b *p*<0.05 for the difference in proportion compared to 1986–1987.

c *p*<0.05 for the difference in proportion compared to 2008–2010.

## Discussion

We observed a decline in the prevalence of both major and minor depression in 85-year-olds between 1986 and 2015, paralleled by a reduction in MADRS scores. The lower odds ratios of depression in later-born cohorts were not fully explained by sociodemographic and health-related factors. The use of antidepressants increased, while the use of anxiolytics and antipsychotics decreased between cohorts. The proportion using any psychotropic drug remained stable across cohorts.

We could identify no published study focusing specifically on octogenarians for comparison. Our results regarding depression in 85-year-olds are in line with previously mentioned studies on older adults in general from Europe (Beller et al., 2021) and the United States (Sullivan et al., 2020; Zivin et al., 2013), all of which found a decreasing trend. Arthur et al. (2020) found no significant reduction in depression prevalence in a British 65 + population between 1990 and 2008. However, confidence intervals for age strata 85 + in that study (5.2–16.7% for 1990 and 4.7–7.8% for 2008) included our point estimates for major depression at similar time points (12.4% in 1986 and 6.9% in 2008), thereby not contradicting our results. Hawthorne et al. (2008) found no change in depression prevalence in an Australian sample aged 70 and above, there was however only 6 years between examinations (1998–2004), which might have been a too short period to detect a change.

Prevalence figures for minor and major depression in the present study are all higher than those reported in age strata 81–87 in an urban Swedish sample examined in Stockholm 2001–2004 (Sjöberg et al., 2017). In that study, 7.9% met DSM-IV criteria for minor depression, and 3.2% for major depression. That is slightly less than half the prevalences in our sample examined in 2008. The Stockholm sample is however limited to one of the most affluent districts in Stockholm, Kungsholmen (Statistics Sweden, 2018), possibly explaining the difference.

The difference in major depression between cohort 2008 and 2015 was not statistically significant in our study, indicating that no change in depression prevalence may have occurred in older adults between those time points. That would parallel results from another Stockholm-based study involving somewhat younger samples (mean age 73 years) examined in 2008 and 2014 (Liang et al., 2018). However, it is also possible that our study was underpowered to detect a difference in prevalence between 2008 and 2015.

The reduction in depression prevalence could not be explained by changes in sociodemographic and health-related risk factors for depression. However, a multitude of changes in environmental exposures across the life span may have contributed to the decline. One example is changes in nutrition; food shortage was more likely at the time of birth for the first cohort (1901–1902), and starvation in utero has been associated with major affective disorders (Brown, van Os, Driessens, Hoek, & Susser, 2000). Low birth weight has been related to late-life depression (Gudmundsson et al., 2011). Further, higher fruit and vegetable intake has been associated with better mental health in older adults (Gehlich et al., 2020; Głąbska, Guzek, Groele, & Gutkowska, 2020), and later-born cohorts of older adults have a higher intake of such foods (Samuelsson et al., 2019). Another possibility is that a decline in the incidence of dementia (Skoog et al., 2017; Wolters et al., 2020) has resulted in a lower proportion of the population with prodromal dementia, which may present as depression (Mirza et al., 2016).

A Dutch study focusing on a younger age group (55–64 years) suggested that changes in depression prevalence were explained by changes in functional dependence and morbidity (Jeuring et al., 2018b). We could not replicate this finding as the cohort effect on depression did not change when multimorbidity and functional dependence were included in the regression model. Our sample was however much older and it has been reported that physical morbidity and depression are more strongly associated in younger age groups (Kessler et al., 2010; Schaakxs et al., 2017). It should also be noted that the list of somatic disorders that we based our multimorbidity variable on is not exhaustive, and changes in other somatic disorders may have explained some of the observed cohort differences in depression.

The marked increase in antidepressant use between the first and second cohorts is in accordance with previous reports (Akincigil et al., 2011; Jorm et al., 2017; Moore et al., 2009). As subgroups with major and minor depression were small, we could only establish a significant increase in the use of antidepressants among participants without ongoing depression. In this group, the use of antidepressants was two-fold higher in 2008 and almost three-fold higher in 2015 compared to 1986. As newer types of antidepressants are considered to have milder side effects in older adults (Wilson & Mottram, 2004), they may be prescribed for relapse prevention after remission from depression to a greater extent than the antidepressants used in the 1980s. Previous research indicates that the increase in antidepressant prescription during the last decades is to a large extent due to higher rates of chronic prescription (Moore et al., 2009). However, as antidepressants have also other indications (e.g. pain and anxiety disorders) and we lack data on indications, we cannot be certain of the reasons for the increased use of antidepressants among persons who did not fulfill the criteria for depression at the time of the research interview.

The increase in the use of antidepressants was paralleled by a marked decrease in the use of anxiolytics and antipsychotics, leaving the proportion using any psychotropic drug unchanged. This underlines the need to consider all drug classes in the study of pharmacoepidemiological trends. The rates of treatment with antidepressants, anxiolytics, and hypnotics by depression status in the later-born cohorts are highly similar to rates found in a Swedish study of persons aged 60 and over, examined between 2001 and 2004 (Karlsson, Johnell, Sigström, Sjöberg, & Fratiglioni, 2016). Use of anxiolytics remained stable between 2008 and 2015 in our study, in contrast to findings from a U.S. study (Maust, Blow, Wiechers, Kales, & Marcus, 2017) that reported an increase in anxiolytic prescriptions (benzodiazepines) in older adults 85 years and over, albeit during a slightly earlier period (2003–2012). This underlines that pharmacoepidemiological trends may be country-specific.

As the reduction in the prevalence of major depression coincided with the increased use of antidepressants, an obvious question concerns the potential causality between these variables. One French study found an association between declining depressive symptomatology and increased use of antidepressants in a cohort of older adults (mean age 75 at first examination) followed longitudinally between 1988 and 1999 (Montagnier et al., 2006). A review of data from Australia, Canada, the United States, and England, involving adults of all ages, found no evidence of an association between increased treatment rates of depression and a reduction in prevalence (Jorm et al., 2017). As causal inferences are not possible in our study design, this question remains unsettled. However, we note that antidepressants are effective in the treatment of late-life depression (Kok, Nolen, & Heeren, 2012), and we were unable to find alternative explanations.

### Strengths and limitations

Despite long time intervals between cohort examinations, we were able to employ identical methods, including diagnostic procedures. The psychiatrist who examined the first cohort supervised those who carried out the examinations in the later-born cohorts. The long period also enabled us to compare psychotropic drug use in 85-year-olds over a period that saw important changes in the types of psychotropic drugs prescribed for older persons with the advent of the second-generation antidepressants and antipsychotics, as well as the Z drugs.

Due to the cross-sectional design, we lacked data on depression status before the examinations. Some of the cases of minor depression identified in our study might actually represent treated cases of major depression in incomplete remission. Further, as this was a single-center study, generalizability may be limited.

Although response rates were similar, we note that a selection bias was at play in the later-born cohorts. Participants in 2008 and 2015 had a higher 3-year survival rate than non-participants, which was not the case in the first cohort. This indicates that the population samples in 2008 and 2015 might have been a healthier selection than in 1986, which might have resulted in an overestimation of cohort differences between the first and latter two cohorts. Furthermore, as in any study of this kind, we cannot exclude the possibility that differences in reporting and rating of depressive symptoms might account for some of the cohort differences. However, inter-rater reliability was substantial. Also, if there were a change in the reporting of symptoms of mental ill-health, we would anticipate it to be in the direction of later-born cohorts more openly acknowledging such symptoms. Furthermore, all participants in 1986 were interviewed at home, while participants in 2008 and 2015 were only offered a home visit if they could not come to the outpatient clinic. Participants in 2008 and 2015 may have been more likely to postpone their research examination if they were currently in a major depressive episode.

Regarding psychotropic drugs, we did not have comparable data on dosage in the three cohorts and can make no inferences regarding potential time trends in the quality of drug treatment. Further, we lack a measure of adherence beyond self-report of medication use. However, a comparative study involving 27 European Union countries found Swedish patients to be the most adherent to antidepressant medication (Lewer, O'reilly, Mojtabai, & Evans-Lacko, 2015).

Given the increased mortality associated with depression (Cuijpers et al., 2014), attention must be paid to the issue of survival bias. The representativeness of the study population is limited to survivors, and persons with depression are less likely to reach the age of 85. It is possible that our observed decline in depression prevalence in octogenarians could to some degree be a result of an increasing trend in younger age groups, which would result in fewer depression-prone individuals surviving to age 85. However, such an explanation seems unlikely, given that depression prevalence may have decreased also in septuagenarians (Rydberg Sterner et al., 2019). Also, the excess mortality associated with depression is reported to have decreased since the 1990's (Jeuring et al., 2018a).

Finally, as most information was self-reported, recall bias may have been an issue, especially in depressed participants, and those with cognitive decline. This should not have influenced the depression prevalences in the different cohorts, but might have distorted findings on associations with depression.

## Supplementary material

For supplementary material accompanying this paper visit https://doi.org/10.1017/S0033291721004335. click here to view supplementary material
