# Supplementary material for: Time trends in depression prevalence among Swedish 85-year-olds: repeated cross-sectional population-based studies in 1986, 2008, and 2015
Source: Psychol Med. 2021 Oct 29;53(6):2456–65. doi: 10.1017/S0033291721004335 (PMC10123839; doi:10.1017/S0033291721004335)
Supplement: Supplementary file 1 [file S0033291721004335sup001.docx]

Supplementary material

| **Table S1. Kappa-values for inter-rater reliability for variables utilized to diagnose major depression, based on 169 dual ratings in the Gothenburg H70 Birth Cohort Studies** | |
| --- | --- |
|  | κ |
| Increased sleep | 0.85 |
| Life-weariness | 0.80 |
| Pessimistic thoughts | 0.75 |
| Reduced appetite | 0.74 |
| Depressed mood | 0.72 |
| Lassitude | 0.71 |
| Indecisiveness | 0.70 |
| Reduced interest | 0.68 |
| Fatigue | 0.66 |
| Concentration difficulties | 0.65 |
| Reduced sleep | 0.65 |
| Reduced speech | 0.55 |
| Depressed mood (observed) | 0.51 |
| Restlessness/agitation | 0.46 |
| Inability to think | 0.43 |
| Distractability | 0.41 |
| Reduced motor activity | 0.39 |
| *Average* | 0.63 |

| **Table S2. Linear regression models of changes in MADRS^a^-scores in three cohorts of 85-year-olds^b^** | | | | | | | | | | | |
| --- | --- | --- | --- | --- | --- | --- | --- | --- | --- | --- | --- |
|  |  |  |  |  |  |  |  |  |  |  |  |
|  | Model 1^c^ | | |  | Model 2^d^ | | |  | Model 3^e^ | | |
|  | B | 95% Confidence interval | |  | B | 95% Confidence interval | |  | B | 95% Confidence interval | |
|  |  | Lower | Upper |  |  | Lower | Upper |  |  | Lower | Upper |
| Cohort 2008 vs 1986 (N=781) | -1.52 | -2.55 | -0.49 |  | -1.45 | -2.52 | -0.38 |  | -1.74 | -2.78 | -0.70 |
| Cohort 2015 vs 1986 (N=669) | -1.44 | -2.02 | -0.87 |  | -1.09 | -1.68 | -0.50 |  | -1.17 | -1.74 | -0.60 |
|  |  |  |  |  |  |  |  |  |  |  |  |
| ^a^Montgomery Åsberg Depression Rating Scale | |  |  |  |  |  |  |  |  |  |  |
| ^b^For inclusion, participants must have Mini Mental State Examination scores ≥24. | | | | |  |  |  |  |  |  |  |
| ^c^Simple linear regression |  |  |  |  |  |  |  |  |  |  |  |
| ^d^Multiple linear regression including: Sex, long-term care facility residence, education beyond mandatory, lost partner within 5 years, no current partner, and feelings of loneliness. | | | | | | | | | | | |
| ^e^Multiple linear regression including factors in model 2, and also: Poor self-rated health, dependence in any activities of daily living, past month pain, and number of somatic diseases.  B=Point changes in MADRS-scores. | | | | | | | | | | | |
